# Residual/recurrent lesions after cold-knife conization for high-grade cervical intraepithelial neoplasia: risk factor analysis and clinical management recommendations

**DOI:** 10.3389/fonc.2025.1645322

**Published:** 2025-08-26

**Authors:** Chenchun Wu, Peng Guo, Pengchong Huang, Lili Guo, Lili Xiong

**Affiliations:** ^1^ Department of Gynecology, The First Affiliated Hospital, and College of Clinical Medicine of Henan University of Science and Technology, Luoyang, Henan, China; ^2^ Department of Plastic Surgery, Xijing Hospital, Air Force Medical University, Xian, Shaanxi, China; ^3^ Department of Burn and Plastic Surgery, The 990th Hospital of the Joint Logistic Support Force, Zhumadian, Henan, China; ^4^ Department of Pathology, First Affiliated Hospital of Henan University of Science and Technology, Luoyang, Henan, China

**Keywords:** cold knife conization, high-grade squamous intraepithelial lesion, human papillomavirus, hysterectomy, postoperative residual/recurrent, risk factors

## Abstract

**Objective:**

This study aims to evaluate the risk factors of residual/recurrent lesions of cervical intraepithelial neoplasia 2/3 (CIN2/3) in patients who underwent cold-knife conization (CKC).

**Methods:**

A total of 976 patients with CIN2/3 who were treated with CKC were retrospectively analyzed. Post-CKC follow-up involved a thin-prep cytology test (TCT) and human papillomavirus (HPV) tests. Residual/recurrent lesions after CKC (RLC) were defined as biopsy-proven CIN2/3 during follow-up, whereas residual lesions identified after a hysterectomy (RLH) were defined as lesions in patients who underwent a hysterectomy 1–6 months after CKC and were diagnosed with CIN 2/3, cervical carcinoma *in situ* or invasive cancer. Univariate analysis and multivariate logistic regression analyses were performed to evaluate the relationship among factors such as age, menopausal status, pregnancy, parity, transformation zone, the height of excision, glandular involvement, persistent HPV infection, HPV infection types (preoperative and postoperative), TCT test (preoperative and postoperative), postoperative margins, and endocervical curettage (ECC) results for RLC and RLH.

**Results:**

During the follow-up period, 152/976 (15.57%) of the patients underwent a hysterectomy and the remaining 824 patients completed their continuous follow-up. Of these, 45/824 (5.46%) were diagnosed with RLC and 53/152 (34.87%) of the patients who underwent a hysterectomy were diagnosed with RLH. RLC was significantly associated with factors such as persistent HPV infection, HPV infection types (preoperative and postoperative), TCT test (6-month postoperative), postoperative margins, and ECC results (*P* < 0.05). Of these variables, persistent HPV infection, HPV 16/18 infection (preoperative), positive margins, TCT test ≥ ASC-US (6- month postoperative), and HPV 16/18 infection (6-month postoperative) emerged as independent risk factors for RLC (*P* < 0.05). In patients undergoing a hysterectomy, RLH was linked to the transformation zone, the height of excision, glandular involvement, HPV infection types (preoperative), TCT test (preoperative), postoperative margins, and ECC results (*P* < 0.05). HPV 16/18 infection (preoperative), transformation zone (type 3), positive margins, and positive ECC appeared to be independent risk factors for RLH (*P* < 0.05).

**Conclusion:**

Risk factors associated with RLC and RLH must be considered when implementing targeted clinical interventions. Elongating the clinical follow-up period is of paramount importance, particularly for patients with high-risk factors; therefore, it is recommended that follow-up intervals be reduced. For patients with HPV16/18 infection, positive margins, and positive ECC, it is recommended that a hysterectomy be performed whenever necessary.

## Introduction

High-grade squamous intraepithelial lesions (HSILs) encompassing cervical intraepithelial neoplasia (CIN) 2/3 have considerable potential for evolving into invasive cervical carcinoma and are closely linked to human papillomavirus (HPV) infection (1, 2). A retrospective study revealed that approximately 31% of CIN3 developed into invasive cancer within 30 years without treatment, albeit this ratio declined to 0.7% with regular treatment (3). Conization is the recommended treatment for HSIL (4). This therapy can be performed using cold-knife conization (CKC), laser conization, loop electro-excision procedure (LEEP), or large-loop excision of the transformation zone (5). LEEP and CKC are the most common surgical procedures conducted in China. LEEP has been demonstrated to be equivalent to CKC in preventing persistent CIN (6). When compared with LEEP, CKC is more complex; however, the rate of residual/recurrent lesions can be controlled at a lower level and the later rate of a hysterectomy can be reduced owing to deep surgical incision (7). Moreover, CKC can provide original tissue specimens that have not been electrothermally burned at the excised margin, thereby aiding the pathological diagnosis (8). As such, CKC is an important method for treating cervical lesions, especially HSIL. Nevertheless, the risk factors associated with residual/recurrent lesions post-CKC warrant elucidation (9). Furthermore, patients who have a pathological upgrade or encounter other complications after CKC may opt for a hysterectomy. Hence, the related risk factors must be investigated, guiding further clinical treatment based on these factors after CKC and hysterectomy (10, 11).

In this study, we aimed to determine the risk factors for RLC and RLH, as well as to develop programmed treatment recommendations based on them.

## Materials and methods

### Patient recruitment

From January 2015 to October 2022, a total of 1236 patients with high-grade CIN underwent CKC treatment at the Department of Obstetrics and Gynecology, First Affiliated Hospital of Henan University of Science and Technology. This study was conducted in accordance with the principles outlined in the Declaration of Helsinki. Our study obtained written informed consent from all participants and was approved by the Ethics Committee of the First Affiliated Hospital of Henan University of Science and Technology (approval number 2024-0553). The age range of the patients varied from 33 to 78 (average: 48.2) years. Patients participating in this study underwent follow-up examinations at 6, 12, 18, and 24 months after surgery, followed by annual assessments thereafter. During each follow-up visit, the participants received a TCT and an HPV test; abnormal results were followed by a colposcopy-guided cervical biopsy (12). The exclusion criteria were as follows: 1. Patients unable to attend follow-up appointments punctually; 2. Patients who could not obtain cytological and HPV test results during their follow-up assessments; 3. Patients diagnosed with cervical cancer at the time of conization. Ultimately, 976 patients were included in this retrospective study, of which 152 patients underwent a laparoscopic hysterectomy within 1–6 months of CKC. The reasons they underwent hysterectomy included: 1. high level of surgical difficulty in performing repeat CKC procedures due to factors such as altered cervical anatomy and unusual lesion location; 2. difficulty accepting strict follow-up regimen and the patient requested hysterectomy; 3. severe psychological distress and significant mental stress caused by concerns about potential invasive cervical cancer, which adversely affects the quality of life; 4. having no fertility requirements, and having other benign uterine diseases such as myoma and adenomyosis. The hysterectomy performed here served both therapeutic and diagnostic purposes.

### CKC

Most patients underwent surgery under spinal, epidural, or general anesthesia. During the operation, the doctor used a scalpel to remove cone-shaped tissues from the cervix. The extent and depth of the resection depended on the lesion depth. The surgical procedure was conducted in adherence to the guidelines outlined in the 2011 edition of the International Federation for Cervical Pathology and Colposcopy (IFCPC). As per these guidelines, for type 1 transformation zones, a complete excision from a depth of 7–10 mm is recommended. For type 2 transformation zones, partial excision of cervical canal tissue is recommended along with the removal of the transformation zone from a depth of 10–15 mm. Deeper excision (15–25 mm) is recommended for type 3 transformation zones owing to incomplete visualization and uncertainty regarding extension into the cervical canal in order to minimize positive margins. As lesions typically involve glandular tissues of ≤ 5 mm depth, it is advisable to limit the thickness of excised tissue to within 7 mm. Specimens were accordingly marked at the 12 O’clock position, and the length and width of each specimen were measured before fixing it. Two senior pathologists verified the pathology reports of the specimens.

### Hysterectomy

In this procedure, following the induction of general anesthesia, a gas pneumoperitoneum was established within the abdominal cavity to typically maintain an intra-abdominal pressure of 12–15 mmHg. Upon introduction of the laparoscope into the abdomen, careful observation of the intra-abdominal organs, including the uterus, ovaries, fallopian tubes, and intestines, was conducted to assess the lesion’s location, extent, and its relationship with the adjacent tissues. Subsequently, dissection of the round ligament, broad ligament, and cardinal ligament was performed to expose the uterine blood vessels. These vessels were then ligated using vascular clamps or sutures to minimize hemorrhage. The uterosacral ligament and cardinal ligaments were also transected for complete removal of the uterus. Postoperative monitoring was essential in such cases to detect any bleeding or fluid accumulation at the incision site while ensuring the patency of drainage tubes (13).

### Histopathological diagnosis

In this study, all diagnoses of CIN1, CIN2/3, or cervical cancer were based on the results of histopathological examination of surgical specimens. The pathology reports described in detail the severity of the lesions, the status of the resection margin, and the degree of glandular involvement in detail. All cases diagnosed as CIN 2/3, cervical carcinoma *in situ* or invasive cancer were confirmed by another pathologist.

### TCT

The TCT serves as a crucial diagnostic tool for the screening of cervical cancer and its precursors. Typically, a doctor uses a speculum to open the vagina to fully expose the cervix. A special cervical brush is then used to gently rotate at the cervical opening and in the cervical canal to collect the exfoliated cervical cells. In this study, the cell preservation solution was processed to remove impurities such as blood and mucus. Fixation, staining, and other operations were then performed on the cell smear to facilitate microscopic observation of the cell morphology microscopically. The final cytological diagnosis was then confirmed by two senior pathologists.

### HPV testing

High-risk HPV (HR-HPV) DNA detection was performed to amplify 37 HPV genotypes, including 13 high-risk types (i.e., types 16, 18, 31, 33, 35, 39, 45, 51, 52, 56, 58, 59, and 68), 5 probable high-risk types (i.e., types 26, 53, 70, 73, and 82), and 18 low-risk and unknown-risk types (i.e., types 6, 11, 34, 40, 42, 43, 44, 54, 55, 57, 61, 67, 69, 71, 72, and CP8304 [HPV-81], 83, and 84). The assay was performed in accordance with the manufacturer’s protocol. Persistent HPV infection refers to the identification of the same high-risk type of HPV in two consecutive tests conducted at a 1-year interval (14, 15).

### The Definition and Time Limit of Residual/Recurrent Lesions

Residual lesion refers to the incomplete removal of the pathological focus of a lesion during surgery, with histologically confirmed persistent lesions within 6 months of operation, often located in the original surgical area. Recurrence is defined as new histological evidence of lesions emerging more than 6 months after surgery, which may appear either at the primary surgical site or at new anatomical locations (16). Lesions detected within 6 months after operation post-CKC are classified as residual lesions, whereas those identified beyond 6 months are considered to be recurrent. Since hysterectomy was performed 1–6 months after CKC, lesions identified through pathological examination following hysterectomy are categorized as residual lesions.

### Statistical methods

SPSS 23.0 statistical software was employed to analyze the data. The chi-square test was applied to conduct a univariate statistical analysis of the risk factors for RLC and RLH. Subsequently, these risk factors that demonstrated correlations (*P* < 0.05) in the univariate analysis were included in the logistic regression analysis. Logistic regression analysis was performed to identify independent risk factors. *P* < 0.05 was considered to indicate statistical significance.

## Results

From January 2015 to October 2022, 1236 patients were diagnosed with CIN2/3 and underwent treatment via CKC at the First Affiliated Hospital of Henan University of Science and Technology. Of these, 260 patients were excluded because of incomplete TCT or HPV DNA follow-up data. Ultimately, 976 patients (mean age: 35.3 ± 9.7 years), with an average follow-up duration of 24.7 months (range: 6–60 months, median 22 months), were included in the study.

During the follow-up period, of the 976 patients, 152 underwent hysterectomy, of whom 53 were pathologically diagnosed with CIN2/3 after the surgery. The remaining 824 patients were followed up after the surgery, and 45 were diagnosed with CIN2/3 during the follow-up period. In this study, the mean interval between CKC surgery and the identification of residual/recurrent lesions was 9.7 months (range 6–48 months, median 12 months). Of the 45 patients diagnosed with CIN2/3 postoperatively, 25 were identified within 1 year, whereas 20 were diagnosed between 1 and 2 years. A total of 152 patients underwent a hysterectomy within 1–6 months following CKC.

In this study, RLC was significantly associated with factors such as persistent HPV infection, HPV infection types (preoperative and postoperative), TCT test (6-month postoperative), postoperative margins, and ECC results (positive ECC ≥CIN2/3) (*P* < 0.05) (**Table 1**). However, age, menopausal status, pregnancy, parity, transformation zone, the height of excision, glandular involvement, and TCT test (preoperative) were not significantly associated with RLC (*P >* 0.05) (**Table 1**).

**Table 1:** Risk factors associated with RLC.

Risk factors	No residual/ recurrent	Residual/ recurrent	*p* value
Age (years)			
≤40	113	4	0.294
>40	666	41	
Menopausal state			
Yes	494	26	0.446
No	285	19	
Pregnancy			
≤1	418	21	0.361
≥2	361	24	
Parity			
≤1	481	33	0.119
≥2	298	12	
Transformation zone			
Type 1	116	7	0.467
Type 2	183	14	
Type 3	480	24	
Height of excision (mm)			
≤15	380	19	0.392
>15	399	26	
Glandular involvement			
Yes	246	19	0.137
No	533	26	
Persistent HPV infection			
Yes	148	15	0.019
No	631	30	
HPV infection types (Preoperative)			
16/18 types	382	31	0.035
Other types	369	13	
Negative	28	1	
TCT test (Preoperative)			
≥ASC-US	664	35	0.175
NOMAL	115	10	
Postoperative margins			
Positive	98	18	<0.001
Negative	681	27	
ECC results			
Positive	204	22	<0.001
Negative	575	23	

ASC-US, Atypical Squamous Cells of Undetermined Significance; *P* < 0.05 indicates a statistically significant difference between the two groups.

During the 6-month follow-up period, 824 patients who had undergone treatment underwent TCT testing. TCT test ≥ASC-US was made in 137 patients, which was confirmed via biopsy and found to be CIN2/3 in 36 patients. The likelihood of residual/recurrent lesion was significantly higher in patients with TCT test ≥ASC-US during the 6-month follow-up (*P* < 0.001) (**Table 2**). In this investigation, 824 patients underwent HPV DNA testing at the 6-month follow-up after CKC, and HPV infection was detected in 210 of the 824 cases. Of these, 35 were pathologically confirmed to have CIN2/3. The likelihood of being diagnosed with CIN2/3 was significantly higher in patients infected with HPV 16/18 than in those who were HPV-negative at the 6-month follow-up (*P* < 0.001) (**Table 2**).

**Table 2:** TCT test and HPV 16/18 infection associated with residual/recurrent lesions in women 6 months after CKC.

Follow-up indicators	No residual/ recurrent	Residual/ recurrent	*p* value
TCT test			
≥ASC-US	101	36	<0.001
Normal	678	9	
HPV 16/18 infection			
Positive	175	35	<0.001
Negative	604	10	

*P* < 0.05 indicates a statistically significant difference between the two groups.

In patients who underwent a hysterectomy, RLH was associated with transformation zone, the height of excision, glandular involvement, HPV infection types (preoperative), TCT test (preoperative), postoperative margins, and ECC results (*P* < 0.05). However, age, menopausal status, pregnancy, and parity were not significantly associated with RLH (*P* > 0.05) (**Table 3**).

**Table 3:** Risk factors associated with RLH.

Risk factors	No residual	Residual	*p* value
Age (years)			
≤50	15	7	0.745
>50	84	46	
Menopausal state			
Yes	63	30	0.397
No	36	23	
Pregnancy			
≤1	52	24	0.395
≥2	47	29	
Parity			
≤1	64	30	0.331
≥2	35	23	
Transformation zone			
Type 1	18	2	0.031
Type 2	36	19	
Type 3	45	32	
Height of excision (mm)			
≤15	59	41	0.028
>15	40	12	
Glandular involvement			
Yes	38	20	0.025
No	61	33	
HPV infection types (Preoperative)			
16/18 types	47	36	0.036
Other types	49	17	
Negative	3	0	
TCT test (Preoperative)			
≥ASC-US	85	51	0.047
Normal	14	2	
Postoperative margins			
Positive	60	43	0.010
Negative	39	10	
ECC results			
Positive	21	20	0.029
Negative	78	33	

*P* < 0.05 indicates a statistically significant difference between the two groups.

Based on the results of univariate analysis, a multivariate logistic regression analysis with adjusted variables was performed to determine the independent high-risk factors for RLC and RLH. Of these variables, persistent HPV infection (odds ratio [OR] 9.237, 95% confidence interval [CI] 1.858–41.519), HPV 16/18 infection (preoperative, OR 7.614, 95% CI 1.345–29.472), positive margins (OR 9.751, 95% CI 1.681–42.886), TCT test ≥ ASC-US (6-month postoperative, OR 14.527, 95% CI 2.373–52.721), and HPV 16/18 infection (6-month postoperative, OR 8.886, 95% CI 1.514–35.459) emerged as independent risk factors for RLC (*P* < 0.05) (**Table 4**). HPV 16/18 infection (preoperative, OR 2.613, 95% CI 1.226–5.569), transformation zone (type 3, OR 2.315, 95% CI 1.112–4.820), positive margins (OR 2.959, 95% CI 1.276–6.866), and positive ECC (OR 2.400, 95% CI 1.044–5.518) were independent risk factors for RLH (*P* < 0.05) (**Table 5**).

**Table 4:** Multivariate logistic regression of risk factors of RLC.

Candidate variables	B	SE	WALD	*P* value	OR	95% Cl
Persistent HPV infection	3.473	1.322	6.898	0.012	9.237	1.858–41.519
HPV 16/18 infection (Preoperative)	1.336	1.084	5.887	0.034	7.614	1.345–29.472
HPV 16/18 infection (6-month postoperative)	2.113	1.052	5.043	0.021	8.886	1.514–35.459
Positive margins	2.387	0.964	6.131	0.011	9.751	1.681–42.886
TCT test≥ASC-US (6-month postoperative)	2.362	1.360	9.797	0.006	14.527	2.373–52.721

Hosmer-Lemeshow test: *χ²=2.261*, *df=6* and *P=0.894*; *P* < 0.05 indicates a statistically significant difference between the two groups.

**Table 5:** Multivariate logistic regression of risk factors of RLH.

Candidate variables	B	Se	WALD	*P value*	OR	95% Cl
HPV 16/18 infection (Preoperative)	0.960	0.386	6.183	0.013	2.613	1.226–5.569
Transformation zone (Type 3)	0.840	0.374	5.037	0.025	2.315	1.112–4.820
Positive margins	1.085	0.429	6.384	0.012	2.959	1.276–6.866
Positive ECC	0.876	0.425	4.252	0.039	2.400	1.044–5.518

Hosmer-Lemeshow test: *χ²=5.948*, *df=8* and *P=0.653*; *P* < 0.05 indicates a statistically significant difference between the two groups.

## Discussion

Past studies have mostly focused on the risk factors for residual/recurrent lesions after cervical cone biopsy (including LEEP and CKC) (17). In contrast, in this study, only those patients who underwent CKC were included so as to reduce the interference of confounding factors. Furthermore, CKC has a lower postoperative infection rate, a more comprehensive cone cut, a lower incidence of residual/recurrent lesions, more accurate pathological examination results, and more precise pathological information, especially for evaluating squamous intraepithelial lesion grading and stromal invasion (18, 19). In CKC, the entire uterus is not removed, which renders the possibility of residual/recurrent lesions after the procedure (20). Long-term meticulous follow-up and postoperative treatment are thus crucial in such cases (21). Moreover, the risk factors for RLC and RLH should be analyzed to provide reasonable suggestions for clinical work-up.

In this study, a follow-up analysis was conducted on 976 patients who received CKC treatment for CIN2/3, with an average follow-up time of 24.7 months. This duration exceeded the follow-up time of most relevant studies, which permitted the detection of residual/recurrent lesions in a comprehensive manner (22). Throughout the follow-up period, TCT testing and HPV infection testing were implemented as a part of the monitoring protocol. TCT test is recognized as the most precise method for diagnosing high-grade intraepithelial neoplasia, whereas HPV infection is acknowledged as a key contributor to precancerous alterations under gynecological conditions (23). Furthermore, we selected a combination of residual and recurrent lesions for analysis, as persistent lesion progression typically follows a continuum that is challenging to strictly delineate at a single time point. Regardless of residual or recurrent status, patients require comparable follow-up strategies (e.g., HPV testing, TCT, and colposcopic biopsy) as well as appropriate therapeutic interventions (e.g., hysterectomy).

Several factors can affect RLC (9). For instance, in our study, RLC was significantly associated with factors such as persistent HPV infection, HPV infection types (preoperative and postoperative), TCT test (6-month postoperative), postoperative margins, and ECC results (*P* < 0.05). Nonetheless, age, menopausal status, pregnancy, parity, transformation zone, the height of excision, glandular involvement, and TCT test (preoperative) were not significantly associated with RLC (*P >* 0.05). Previous studies suggest persistent HPV infection as an important risk factor for RLC, which was confirmed in this study through a long-term follow-up. During the follow-up period, especially in patients diagnosed with HPV16/18 infection, the risk of developing RLC was higher. Consistent with other studies, women with positive margins exhibited a relatively higher risk of recurrence (24, 25). In addition, TCT test ≥ASC-US (6-month postoperative) has been reported to be a possible risk factor that necessitates further exploration for residual/recurrent cases (26). To the best of our knowledge, this is the first study to investigate the correlation between preoperative ECC results and residual/recurrent lesions during follow-up. A positive ECC result indicates deeper penetration of the lesion into the cervical canal, which substantially increases the risk of recurrence and is statistically significant. To assess the independent risk factors for RLC, a multivariate logistic regression analysis was conducted. The findings indicated that persistent HPV infection, HPV 16/18 infection (preoperative), positive margins, TCT test ≥ASC-US (6- month postoperative), and HPV 16/18 infection (6-month postoperative) were independent risk factors for RLC.

In patients undergoing a hysterectomy after CKC, RLH was associated with the transformation zone, the height of excision, glandular involvement, HPV infection types (preoperative), TCT test (preoperative), postoperative margins, and ECC results (*P* < 0.05). HPV 16/18 infection (preoperative), transformation zone (type 3), positive margins, and positive ECC as independent risk factors.

TCT is necessary for preoperative examination and postoperative follow-up and is related to the initial diagnosis and the selection of surgical approach for patients. The findings of this study indicated that TCT test ≥ASC-US (preoperative) is a risk factor for RLH in patients who have undergone a hysterectomy. This result signifies that the higher the malignancy grade diagnosed preoperatively, the greater is the risk of RLH (27, 28). Qing reported that a cervical excision length of 10–15 mm is reasonable for patients with TZ1 and TZ2, whereas 17–25 mm is optimal for TZ3 excision with more negative internal margins (5). The type of TZ2 and TZ3 indicates whether the cervical transformation zone is partly visible or not visible. Therefore, our results suggest that the transformation zone and the height of the excision are the risk factors that prompt further examinations for residual lesions. Kang observed that women infected with HPV16 and HPV18 are more likely to have residual lesions after LEEP (29). Our results also revealed that positive HPV16/18 status is an independent predictive factor for RLH. In line with numerous other studies, our research asserted that a positive margin is an independent risk factor for RLH. Furthermore, it is worth noting that among patients with positive margins who underwent a hysterectomy, the proportion of pathologically confirmed nonresidual lesions were 58.25% (60/103). Hence, we contend that a total hysterectomy is not an optimal choice merely because the margins are positive. Our observations imply that positive ECC are independent risk factors for RLH, which are related to the fact that they suggest the presence of abnormal cells or pathological tissues within the cervical canal. These pathologies may include CIN, especially high-grade CIN, or even cervical cancer. Similarly, glandular involvement signifies the infiltration of the lesional tissue into the subepithelial glandular structures, contributing to the incomplete excision of the lesion and, consequently, increased risk of residual/recurrent lesions. Our findings revealed a significant association between glandular involvement and RLH, indicating that pathological evidence of glandular involvement after hysterectomy is an important factor influencing residual lesions. However, logistic regression analysis demonstrated that glandular involvement was not an independent risk factor for RLH, suggesting that its impact on RLH loses independence when comprehensively analyzed alongside other factors, potentially being masked or confounded by them.

According to the guidelines of the American Society for Colposcopy and Cervical Pathology and the National Comprehensive Cancer Network in the United States, patients with positive margins following initial cone biopsy may opt for close follow-up, undergo further diagnostic procedures for cervical lesions, or proceed to total hysterectomy (30). In our research, of the 824 patients who were followed up, 116 presented with positive resection margins. Of these 116 patients, 18 (15.52%) exhibited residual/recurrent lesions. Of the 152 patients who underwent a hysterectomy, 103 showed positive margins before the operation, and 43 (41.75%) of these 103 patients (41.75%) had residual lesions. Positive margins act as independent risk factors for RLC and RLH. However, the proportion of patients who ultimately developed CIN2/3 in both groups of patients with positive margins was <50%. Consequently, the treatment plan and surgical approach for patients could not be determined merely based on this single factor.

Our study specifically focused on patients who underwent CKC treatment, thereby minimizing confounding interference from different surgical approaches in risk factor assessment. Additionally, this investigation comprehensively evaluated RLC and RLH patients, enhancing the precision and inclusiveness of clinical decision-making. However, our study is limited by its retrospective design and the single-center nature, as well as a lack of data related to preoperative and follow-up HPV viral load and the extended follow-up after CKC treatment. Furthermore, considering that this is a retrospective study, postoperative immunohistochemical outcomes, such as the expression of Ki-67 and P16, were unavailable, which may also function as risk factors for recurrent or residual lesions (31, 32).

In conclusion, our findings suggest that patients should be managed in a stratified manner, with those having high-risk factors requiring more frequent follow-ups with shorter intervals to mitigate the risk of recurrence: an initial follow-up at 3 months postoperatively (combining TCT and HPV testing), followed by assessments every 3–6 months (for at least 2 years). After 2 years, if results remain persistently negative, the interval may be extended to annually. Whether patients require additional surgery should also be dealt with differently. Those with high-risk factors, no fertility desires, and advanced age can opt for a hysterectomy. This integrated management approach can contribute to establishing a comprehensive treatment plan and alleviating patients’ anxiety.

## Data Availability

The original contributions presented in the study are included in the article/supplementary material. Further inquiries can be directed to the corresponding author.

## References

[1] Sung H Ferlay J Siegel RL Laversanne M Soerjomataram I Jemal A. Global cancer statistics 2020: GLOBOCAN estimates of incidence and mortality worldwide for 36 cancers in 185 countries. CA Cancer J Clin. (2021) 71:209–49. doi: 10.3322/caac.21660, PMID: 33538338

[2] Palumbo M Della Corte L Ronsini C Guerra S Giampaolino P Bifulco G. Surgical treatment for early cervical cancer in the HPV era: state of the art. Healthcare (Basel). (2023) 11:2942. doi: 10.3390/healthcare11222942, PMID: 37998434 PMC10671714

[3] Petry KU. Management options for cervical intraepithelial neoplasia. Best Pract Res Clin Obstet Gynecol. (2011) 25:641–51. doi: 10.1016/j.bpobgyn.2011.04.007, PMID: 21723198

[4] Chen JY Wang ZL Wang ZY Yang XS. The risk factors of residual lesions and recurrence of the high-grade cervical intraepithelial lesions (HSIL) patients with positive-margin after conization. Med (Baltimore). (2018) 97:e12792. doi: 10.1097/MD.0000000000012792, PMID: 30313104 PMC6203583

[5] Cong Q Yu Yi Xie Yu Li Y Sui L. Risk factors of LEEP margin positivity and optimal length of cervical conization in cervical intraepithelial neoplasia. Front Oncol. (2023) 13:1209811. doi: 10.3389/fonc.2023.1209811, PMID: 37427119 PMC10324658

[6] Wu Q Jiang Y Ding J Xia L Xu H. Clinical predictors of residual disease in hysterectomy following a loop electrosurgical excision procedure for cervical intraepithelial neoplasia grade 3. BMC Pregnancy Childbirth. (2022) 22:971. doi: 10.1186/s12884-022-05281-y, PMID: 36575366 PMC9793502

[7] Santesso N Mustafa RA Wiercioch W Kehar R Gandhi S Chen Y. Systematic reviews and meta-analyses of benefits and harms of cryotherapy, LEEP, and cold knife conization to treat cervical intraepithelial neoplasia. Int J Gynecol Obstet. (2016) 132:266–71. doi: 10.1016/j.ijgo.2015.07.026, PMID: 26643302

[8] Jiang Y Chen C Li L. Comparison of cold-knife conization versus loop electrosurgical excision for cervical adenocarcinoma *in situ* (ACIS): A systematic review and meta-analysis. PloS One. (2017) 12:e0170587. doi: 10.1371/journal.pone.0170587, PMID: 28125627 PMC5268480

[9] Arbyn M Redman C Verdoodt F Kyrgiou M Tzafetas M Ghaem-Maghami S. Incomplete excision of cervical precancer as a predictor of treatment failure: a systematic review and meta-analysis. Lancet Oncol. (2017) 18:1665–79. doi: 10.1016/S1470-2045(17)30700-3, PMID: 29126708

[10] Scherer-Quenzer AC Findeis J Herbert SL Yokendren N Reinhold AK Schlaiss T. The value of endocervical curettage during large loop excision of the transformation zone in combination with endocervical surgical margin in predicting persistent/recurrent dysplasia of the uterine cervix: a retrospective study. BMC Womens Health. (2024) 24:461. doi: 10.1186/s12905-024-03291-w, PMID: 39169335 PMC11337847

[11] Wang J Wang C Su T. Risk factors for residual lesions after total hysterectomy in patients with high-grade cervical intraepithelial neoplasia. BMC Womens Health. (2024) 24:369. doi: 10.1186/s12905-024-03212-x, PMID: 38915002 PMC11194937

[12] Kong TW Son JH Chang SJ Paek J Lee Y Ryu HS. Value of endocervical margin and high-risk human papillomavirus status after conization for high-grade cervical intraepithelial neoplasia, adenocarcinoma in *situ*, and microinvasive carcinoma of the uterine cervix. Gynecol Oncol. (2014) 135:468–73. doi: 10.1016/j.ygyno.2014.09.022, PMID: 25284039

[13] Kesic V Dokic M Atanackovic J Milenkovic S Kalezic I Vukovic S. Hysterectomy for treatment of CIN. J Low Genit Tract Dis. (2003) 7:32–5. doi: 10.1097/00128360-200301000-00008, PMID: 17051042

[14] Przybylski M Pruski D Millert-Kalinska S Zmaczynski A Baran R Horbaczewska A. Remission of HPV infection after LEEP-conization - a retrospective study. Ginekologia Polska. (2022) 93:99–104. doi: 10.5603/GP.a2021.0164, PMID: 35072214

[15] Visioli CB Iossa A Gorini G Mantellini P Lelli L Auzzi N. The 5-year risk of recurrence of grade 2/3 cervical intraepithelial neoplasia after treatment in a population screening program by human papillomavirus status: A cohort study in central Italy. J Med Screen. (2023) 30:191–200. doi: 10.1177/09691413231175630, PMID: 37229655

[16] Duesing N Schwarz J Choschzick M Jaenicke F Gieseking F Issa R. Assessment of cervical intraepithelial neoplasia (CIN) with colposcopic biopsy and efficacy of loop electrosurgical excision procedure (LEEP). Arch Gynecol Obstet. (2012) 286:1549–54. doi: 10.1007/s00404-012-2493-1, PMID: 22865036

[17] Ding T Li L Duan R Chen Y Yang B Xi M. Risk factors analysis of recurrent disease after treatment with a loop electrosurgical excision procedure for high-grade cervical intraepithelial neoplasia. Int J Gynecol Obstet. (2023) 160:538–47. doi: 10.1002/ijgo.14340, PMID: 35810389 PMC10087663

[18] El-Nashar S Shazly S Hopkins M Bakkum-Gamez JN Famuyide AO. Loop electrosurgical excision procedure instead of cold-knife conization for cervical intraepithelial neoplasia in women with unsatisfactory colposcopic examinations: A systematic review and meta-analysis. J Low Genit Tract Dis. (2017) 21:129–36. doi: 10.1097/LGT.0000000000000287, PMID: 27977541

[19] Li X Liu M Ji Y Qu P. The effectiveness of cold-knife conization (CKC) for post-menopausal women with cervical high-grade squamous intraepithelial lesion: a retrospective study. BMC Surg. (2021) 21:241. doi: 10.1186/s12893-021-01238-8, PMID: 33975589 PMC8114500

[20] Kim YT Kim JW Kim DK Song CH. Loop diathermy and cold-knife conization in patients with cervical intraepithelial neoplasia: a comparative study. J Korean Med Sci. (1995) 10:281–6. doi: 10.3346/jkms.1995.10.4.281, PMID: 8593209 PMC3054059

[21] Lei L Zhang L Zheng Y Ma W Liu F Li D. Clinical analysis of 314 patients with high-grade squamous intraepithelial lesion who underwent total hysterectomy directly: a multi-center, retrospective cohort study. BMC Cancer. (2024) 24:575. doi: 10.1186/s12885-024-12342-2, PMID: 38724921 PMC11080298

[22] Serati M Siesto G Carollo S Formenti G Riva C Cromi A. Risk factors for cervical intraepithelial neoplasia recurrence after conization: a 10 year study. Eur J Obstet Gynecol Reprod Biol. (2012) 165:86–90. doi: 10.1016/j.ejogrb.2012.06.026, PMID: 22771223

[23] Zhong G Wang Y Yao S Fang X Lin R Lin Z. Squamous cell carcinoma antigen combined with HPV-16 infection in predicting high-grade squamous intraepithelial lesions of the cervix. J Obstet Gynecol. (2022) 42:696–700. doi: 10.1080/01443615.2021.1948511, PMID: 34565271

[24] Aguiar TD Valente RP Figueiredo AR Beires JM Vieira-Baptista P. Risk factors for positive margins in high-grade cervical intraepithelial neoplasia after transformation zone excision. J Low Genit Tract Dis. (2022) 26:207–11. doi: 10.1097/LGT.0000000000000668, PMID: 35314587

[25] Ørbo A Arnesen T Arnes M Straume B. Resection margins in conization as prognostic marker for relapse in high-grade dysplasia of the uterine cervix in northern Norway: a retrospective long-term follow-up material. Gynecol Oncol. (2004) 93:479–83. doi: 10.1016/j.ygyno.2004.03.010, PMID: 15099966

[26] Wu J Jia Y Luo M Duan Z. Analysis of residual/recurrent disease and its risk factors after loop electrosurgical excision procedure for high-grade cervical intraepithelial neoplasia. Gynecol Obstet Invest. (2016) 81:296–301. doi: 10.1159/000437423, PMID: 26337009

[27] Dimitrios P Martyn U William P. Endocervical crypt involvement by high-grade cervical intraepithelial neoplasia and its association with high-grade histopathological recurrence after cervical excision in women with negative excision margins: a systematic review and meta-analysis. Arch Gynecol Obstet. (2024) 309:939–48. doi: 10.1007/s00404-023-07242-y, PMID: 37821642 PMC10867046

[28] Zeng Y Jiang T Zheng Y Yang J Wei H Yi C. Risk factors predicting residual lesion in subsequent hysterectomy following cold knife conization (CKC) for high-grade squamous intraepithelial lesion (HSIL). BMC Womens Health. (2022) 22:358. doi: 10.1186/s12905-022-01939-z, PMID: 36042513 PMC9426006

[29] Kang WD Oh MJ Kim SM Nam JH Park CS Choi HS. Significance of human papillomavirus genotyping with high-grade cervical intraepithelial neoplasia treated by a loop electrosurgical excision procedure. Am J Obstet Gynecol. (2010) 203:72. doi: 10.1016/j.ajog.2010, PMID: 20417477

[30] Wright TC Jr Massad LS Dunton CJ Spitzer M Wilkinson EJ Solomon D. 2006 consensus guidelines for the management of women with cervical intraepithelial neoplasia or adenocarcinoma in situ. J Low Genit Tract Dis. (2007) 11:223–39. doi: 10.1016/j.ajog.2007.07.050, PMID: 17917567

[31] Menon SS Guruvayoorappan C Sakthivel KM Rasmi RR. Ki-67 protein as a tumor proliferation marker. Clin Chim Acta. (2019) 491:39–45. doi: 10.1016/j.cca.2019.01.011, PMID: 30653951

[32] Liggett WH Jr Sidransky D. Role of the p16 tumor suppressor gene in cancer. J Clin Oncol. (1998) 16:1197–206. doi: 10.1200/JCO.2016.71.0012, PMID: 9508208

